# Empowering students: positive higher education strategies

**DOI:** 10.3389/fpsyg.2025.1561267

**Published:** 2025-09-19

**Authors:** Honghuan Li

**Affiliations:** School of Foreign Studies, China University of Political Science and Law, Beijing, China

**Keywords:** positive education, higher education, self-determination theory, PERMA framework, PROSPER framework, study demands-resources theory, wellbeing-centered pedagogies, artificial intelligence

## Abstract

Student wellbeing in higher education has been increasingly recognized as a necessary companion to academic achievement. This narrative review examines positive education interventions through the frameworks of Self-Determination Theory (SDT), the PERMA framework, the PROSPER framework, and the Study Demands–Resources (SD-R) Theory, which highlight the importance of autonomy, competence, relatedness and holistic wellbeing in promoting engagement, resilience and intrinsic motivation. The research discusses practical strategies such as mindfulness, strengths-based learning, and growth mindset cultivation and their alignment with institutional policies and emerging technologies for personalized learning. It also identifies challenges such as scalability, cultural adaptation and equity by providing actionable recommendations for building supportive, wellbeing-centered learning environments.

## 1 Introduction

There is a growing recognition in higher education that the mission need extend beyond the cultivation of academic skills to include shaping the outcome of holistic student development and wellbeing (Seligman et al., 2009). With the prevalence of anxiety and depression among university students worldwide, attention to student mental health is on the rise to include an approach on how universities worldwide could maximize learning outcomes and improve psychosocial wellbeing. In this regard, positive education has emerged as an integration of academic learning with positive psychology, in order to empower learners and enhance their personal development (Li, 2025; Oades et al., 2011).

One of the primary theoretical underpinnings of positive education is Self-Determination Theory (SDT) which suggests that human motivation and wellbeing depend on the satisfaction of three basic psychological needs: autonomy, competence, and relatedness (Deci and Ryan, 2000). In the context of higher education, promoting autonomy in students involves providing learning opportunities where students feel they have choice and voice in their academic activities, which would enhance their intrinsic motivation (Ryan, 2017).

Autonomy-supportive climates are also vital to engagement and resilience for the ability to adjust and succeed in the face of challenges (Furlong et al., 2014). In fact, resilience and engagement are now considered key drivers of academic achievement in the short term, as well as personal development in the long term (Martin and Marsh, 2009). The ongoing development of wellbeing-oriented pedagogies indicates a bright future for positive higher education (Oades et al., 2011). While universities strive to integrate wellbeing into institutional policies, curricula and student support services, educators have initiated various interventions, such as mindfulness programs (Goyal et al., 2014) and strengths-based instruction (Seligman, 2011), by perceiving student wellbeing as an evolving goal rather than a byproduct (Kern et al., 2015).

This narrative review aims to examine positive education strategies in the context of prominent theoretical frameworks—specifically SDT, the PERMA framework, the PROSPER framework, and the SD-R theory—and to evaluate their relevance and contribution to positive higher education. Strategies of positive higher education, including mindfulness-based programs, strengths-based education, and growth mindset development, are discussed alongside the key theoretical frameworks that support student autonomy, engagement, and resilience. The review further addresses the integration of wellbeing into institutional policies and the potential of emerging technologies to enhance student support. By linking theory to practice, the focus is on how positive education frameworks can be translated into concrete interventions and policy initiatives that empower students in higher education.

## 2 Methodology

### 2.1 Research method

This review adopted a narrative review approach for its capacity to provide a holistic synthesis of existing literature, unconstrained by the strict protocols of a systematic review (Baumeister and Leary, 1997). This approach allowed us to integrate theoretical constructs, empirical evidence, and practical strategies pertaining to positive education in higher education.

Using a narrative review approach, this research explores positive education strategies in higher education, including the theoretical frameworks, implementation strategies, and its impact on student wellbeing. The review process was: Literature Identification → Categorization → Themes → Results. Two main research questions were articulated in this research:

1. What are the main theories or frameworks relating to positive education?
2. What are the strategies of implementing positive education to empower students in higher education?

### 2.2 Data collection

Relevant studies were identified via systematic searches in peer-reviewed academic journals, books and conference proceedings. Database searches (such as Google Scholar, Web of Science, and Scopus) were conducted to identify relevant sources on positive education interventions, student engagement, positive education frameworks, and the integration of wellbeing into educational policy.

The inclusion criteria were:

1. Studies that discuss theoretical frameworks, empirical studies, or institutional studies related to positive education.
2. Literature addressing the integration of digital tools in wellbeing-centered education.
3. Studies providing cross-cultural perspectives on positive education in higher education settings.

Exclusion criteria included:

1. Studies that focused solely on K-12 education without implications in higher education.
2. Research lacking clear theoretical support in wellbeing-centered pedagogies.
3. Studies with a narrow focus on clinical psychology or medical interventions unrelated to educational contexts.

### 2.3 Data analysis and synthesis

A thematic analysis was used to synthesize themes on strategies, challenges, benefits and trends relating to positive education. The literature was organized into four key themes: (1) Theoretical Foundations—Exploring the fundamental theories or frameworks upon which positive education is based. (2) Intervention Strategies—Specifically targeted programs, e.g., mindfulness training, strengths-based learning, growth mindset cultivation, AI-driven student support systems. (3) Institutional Integration—Understanding how universities integrate positive education into curriculum design, faculty development training, and administrative policies. (4) Barriers to Implementation—Discussing issues such as scalability, cultural sensibility, and the ethics of trending technologies.

## 3 Theories and frameworks of positive education

### 3.1 Self-determination theory

Self-Determination Theory (SDT) is a pivotal in positive psychology. It suggests that general wellbeing and excellent performance are met by three basic human needs: autonomy, competence, and relatedness (Deci and Ryan, 2000). Autonomy refers to the feeling of volition and personal agency over one’s actions (Deci and Ryan, 2000). Within educational settings, autonomy can be facilitated by interventions such as open projects, elective courses, or self-managed projects (Reeve and Tseng, 2011). Students are more engaged in materials, experience more positive academic emotions and exert more effort or persist more in the face of academic setback when they feel their choices are important (Vansteenkiste et al., 2004).

Competence involves feeling that one’s behaviors are effective in a given context (Deci and Ryan, 2000). Providing appropriately challenging tasks, clearly delineated learning objectives, and constructive and timely feedback through coursework can support students’ sense of competence (Reis et al., 2000). Scaffolded assignments building on previous knowledge allow students to gain mastery and confidence, further fueling their internal drive to succeed academically (Ryan, 2017). Relatedness refers to the need to feel socially connected and supported (Baumeister and Leary, 1995). In higher education, there are many ways to facilitate relatedness such as use of group projects, peer mentoring, or discussion forums—methods that engage collaboration and combine students to develop a sense of community (Miserandino, 1996). Many students do not have strong social support on campus and have limited resources for seeking help when needed; thus, the experience of having strong relational connections on campus can be used to alleviate feelings of isolation, relieve stress and promote social and academic development (Furlong et al., 2014).

The core of SDT is the idea that institutional climates can support or hinder student autonomy (Reeve, 2016). Instead, autonomy-supportive teaching strategies emphasize providing rationale, choice and opportunities for self-initiated learning, rather than extrinsic rewards or coercive control (Reeve and Jang, 2006). For example, educators who allow students to participate in curriculum design (e.g., deciding which topics to discuss or customizing research projects) frequently observe increased interest, enthusiasm, and depth of learning (Reeve, 2016). An autonomy-supportive stance also includes empathic listening, taking the perspective of the students into account, and providing constructive guidance instead of directive feedback (Jang et al., 2010). Such an environment would draw on factors such as academic success, but also be associated with better mental health outcomes, such as reduced stress and improved subjective wellbeing (Vansteenkiste et al., 2004). Providing students with the freedom to explore new ideas enables an environment of self-directed learning for students, where they begin to develop a sense of ownership over both personal and professional growth.

Autonomy is closely related to student wellbeing, reflecting positive emotions, life satisfaction, and a sense of meaning in academic pursuits (Ryan, 2017). Autonomy-supportive behaviors may improve resilience by reducing externally imposed pressure and perfectionism through the promotion of choice and self-directed goals (Vansteenkiste et al., 2004). In addition, studies show that more autonomy in education predicts more engagement and intrinsic motivation, which allows for deeper learning and better performance (Jang et al., 2010). Motivated students are more likely to share their ideas in class discussions, work together in group projects, and stick with their studies—all important variables for both short-term achievement and long-term growth (Martin and Marsh, 2009). Hence, strategies fostering autonomy can act as catalysts for holistic student development, uniting academic prowess with socio-emotional thriving.

However, creating autonomy-supportive learning environments can be quite difficult, despite the clear benefits. Faculty may not be experienced with student-centered pedagogies and require professional development in order to effectively create and implement such activities (Reeve, 2016). In addition, strictly large class sizes, heavy teaching loads or limited time may hinder the potential of enriching personalized feedback and continuous dialog (Jang et al., 2010). Nevertheless, these challenges highlight the necessity for flexible, context-responsive solutions that remain faithful to the principles of SDT but also tailor to the richness of the educational contexts in which they are applied (Ryan, 2017). Wehmeyer et al. (2021) describe several interventions derived from SDT—such as training teachers to be more autonomy-supportive and implementing schoolwide practices emphasizing autonomy, competency, and relationships—that have improved student agency and self-directed. Such approaches underscore the importance of promoting student ownership, meaningful engagement, and purpose, aligning with the broader goals of positive education.

### 3.2 PERMA framework

The PERMA model, developed by Seligman (2011), outlines the 5 building blocks of a life of wellbeing: Positive Emotion, Engagement, Relationship, Meaning, and Accomplishment. This model posits that human beings thrive when experiencing a balance between the Pleasant Life (hedonic wellbeing and happiness) and the Meaningful Life (eudaimonic wellbeing). A harmony between these aspects, in turn, mediates an individual’s general state of health and capacity for flourish. In higher education, this idea is predicated on the belief that supporting positive student emotions, learning engagement, social connections, meaning and purpose, and achievement will holistically lead to positive academic and life outcomes.

Within the context of positive education, the PERMA model is utilized by educational institutions as a tool to support both student’s wellbeing and their academic performance. Positive education focuses on the skill of happiness and well-being alongside traditional academic knowledge (Seligman et al., 2009). Character strengths identification, gratitude exercises, and resilience training are included in university curricula, thus taking a holistic approach to student development that exceeds academic knowledge alone (Kern et al., 2015; Kovich et al., 2023; Waters, 2011). Studies show that PERMA interventions increase student engagement and decrease stress. For example, Hendriks et al. (2020) indicated that multi-component Positive Psychology Interventions were more effective in promoting well-being outcomes of students than single-component interventions. This is consistent with the view that a holistic position has the most positive outcomes, particularly in the face of academic demands and students’ often challenging transitions to university (Oades et al., 2011).

In the domain of higher education, Kern et al. (2015) constructed a multidimensional PERMA survey for students, confirming that the five PERMA constructs were meaningful in the university. Kovich et al. (2023) also reported that all five constituents of PERMA consistently emerged in data from undergraduate students and that they loaded onto a higher order well-being factor. These findings provide support for PERMA as a theoretical and measurement framework for the wellbeing of college students. In addition, several PERMA-based programs have had a positive impact. Morgan and Simmons (2021) created an 8-week positive education online program based on PERMA, designed for university students. Furthermore, Dorri Sedeh and Aghaei (2024) conducted a six-session positive education program for undergraduate students on the grounds of the Seligman’s PERMA model. Such studies indicate that teaching and practicing PERMA can result in significant improvements in student mental well-being and robustness.

However, leveraging a broad framework such as the five pillars can be difficult to implement entirely within a university setting. Furthermore, interventions that apply a PERMA framework may require customization for the particular context by which “Meaning” and “Engagement” are constituted for students, which could differ among cultures and academic fields. Maintaining a balance between positive emotion and meaning creates a strong foundation for students to flourish both during their university years and beyond.

### 3.3 PROSPER framework

The PROSPER framework, proposed by Noble and McGrath (2015), involves seven foundational domains: Positivity, Relationships, Outcomes, Strengths, Purpose, Engagement and Resilience. Its purpose is to develop enabling institutions: promoting academic success and mental health through evidence-based practices. The PROSPER framework is based on Seligman’s PERMA model which highlights Positive Emotion, Engagement, Relationships, Meaning, and Accomplishment. PROSPER, however, incorporates two further, crucial dimensions, Strengths and Resilience, whose recognition is burgeoning as being central to wellbeing (Huppert and So, 2013). Strengths are deemed as fundamental in self-development whereas resilience covers one’s ability to adjust when faced with challenging times. The PROSPER framework serves as an organizing structure primarily for schools to implement positive education, overlapping social–emotional learning (SEL) goals with academic development. Each letter of PROSPER corresponds to a domain of practice. For example, Positivity involves fostering positive emotions and attitudes (e.g., gratitude exercises, a supportive and safe learning climate). Relationships emphasizes positive peer and teacher–student relationships to create a sense of belonging, which is associated with lower anxiety and better academic outcomes (Bizumic et al., 2009; Osterman, 2000).

The Outcomes part focuses on goals setting and mastering. Using evidence-based teaching strategies and encouraging students to develop a growth mindset are the key for students attaining academic success and personal fulfillment (Dweck, 2006). In the similar vein, the Strengths dimension promotes the recognition and utilization of both individual and collective strengths, nurturing a sense of competency and purpose (Govindji and Linley, 2007). The practical aspect of the PROSPER framework has been endorsed by both educators and researchers. Educators expressed significant agreement on its usefulness as a common language for wellbeing, and as an instrument for informing best practice (Noble and McGrath, 2015). In addition, aspects of the framework have been successfully implemented in programs such as “Bounce Back” which aims to foster resilience and positive campus culture through curriculum design and relational strategies (McGrath and Noble, 2011).

While the PROSPER framework provides a robust model to implement positive education, it also has shortcomings. It in turns raises a criticism arguing for more extensive empirical examinations in order to study its long-term impact and effectiveness in other educational scenario (Noble and McGrath, 2015). While it is important to focus on policies around wellbeing, it is equally important to mitigate any inequities in access to wellbeing programs so that all students, regardless of socioeconomic background can access and benefit from wellbeing programs. The PROSPER has been a promising step in the direction of realizing the potential for Positive Psychology to be meaningful in education.

### 3.4 Study demands–resources theory

The Study Demands–Resources (SD-R) model was based on the Job Demands–Resources (JD-R) model from psychology (Demerouti et al., 2001), and was proposed as a conceptual model to study student wellbeing through the concepts of study demands and study resources. Study demands are like an academic task that not only require a substantial effort but also generate additional costs for the individual in the long run (Lesener et al., 2020). Study resources, however, are described as the physical, psychological or organizational features that assist students in making responses to those demands that are conducive to successful outcomes. Two general processes are proposed by SD-R theory to influence student wellbeing; one is the health impairment process, and the other is the motivational process (Bakker et al., 2023).

The process of health impairment entails the negative impact of high studying demands such as class demands, complex attributions, and exams, that can deplete students and result in fatigue, stress, and burnout (Madigan and Curran, 2021). Symptoms of burnout, like cynicism and incompetence, reflect negatively on academic outcomes (Salmela-Aro and Upadyaya, 2014), leading to absenteeism, lower grades, and dropout intentions. Conversely, the motivational process underscores the facilitating effect of study resources—e.g. social support, independence, feeding back—on student engagement, which is characterized by vigor, dedication and absorption (Bakker et al., 2015; Schaufeli and Bakker, 2004).

Not all study demands are damaging; some represent hindrances and others represent challenges. Hindrance demands (e.g., ambiguous assignments, competing deadlines), hinder learning and development, hence increasing stress and disengagement (Martin et al., 2023). On the other hand, higher order (challenging, yet clear tasks) challenge demands can induce growth and learning in the presence of adequate resources (Lesener et al., 2020). Students are better able to cope with demands and remain motivated when supported by resources such as teacher support, peer collaboration and access to quality materials (Mokgele and Rothmann, 2014).

SD-R theory also posits two interaction effects: the buffer hypothesis and the boost hypothesis. The buffer hypothesis states that resources can compensate (buffer) for the adverse effect that demands have on health. For example, supportive communication from faculty and/or peers can serve as a buffer to stress from a heavy academic load, such that collaborators have reduced risk for the development of burnout (Aloia and McTigue, 2019). In contrast, the boost hypothesis suggests that resources as challenges will have more positive effects on engagement and performance when more resources are available, and that the level of resources will facilitate the impact of demands on the level of engagement and performance even more (Bakker et al., 2023).

The application of SD-R in higher education thus involves attending to the impact of university structures and teaching practices on the balance of demands and resources in students’ lives. Student-initiated strategies (e.g., study crafting, actively redesigning the methods to study or one’s study environment, and seeking feedback) may also influence study anxiety. These resources allow students to customize their learning, thus make better use of available resources, and thus result in better engagement and performance (Körner et al., 2023). In contrast, maladaptive responses (e.g., procrastination, avoidance) may amplify the impact of high demands, resulting in a vicious cycle of even more stress and even lower performance (Bakker and Costa, 2014).

The SD-R framework offers a valuable perspective for designing actionable models to address the engagement–burnout dynamic. By identifying where demands can be managed or reframed, and how resources can be bolstered (through faculty support, peer mentoring, improved feedback, etc.), institutions can intervene more systematically to improve student wellbeing and academic outcomes.

## 4 Positive education strategies in higher education

### 4.1 Positive education interventions

Positive education refers to the application of positive psychology principles, the science of happiness, resilience, strengths, and optimal functioning, within the educational context (Seligman et al., 2009). Positive education initially focused on K–12 contexts (Waters, 2011), but universities have since recognized the value of incorporating positive interventions across their curricula and student support services, considering their beneficial effects on academic engagement and emotional wellbeing (Oades et al., 2011). Such strategies are typically developed to cultivate resilience—the ability to recover from academic and personal failures—and prolonged engagement, an essential requirement for transcending learning and endurance in tertiary education settings (Martin and Marsh, 2009).

Mindfulness, usually characterized as the nonjudgmental awareness of the current moment (Kabat-Zinn, 2003), is a popular strategy among positive education interventions. A mindfulness-based program in higher education typically includes brief, structured meditation sessions, breathing exercises, reflective journaling, either incorporated into class activities or delivered in co-curricular workshops (Bamber and Kraenzle Schneider, 2016). These programs can achieve the following aspects: (1) Alleviate stress and anxiety: By practicing mindfulness, students indicate feeling more relaxed and develop perspectives under stress (Conley et al., 2016); (2) Improve cognitive performance: Better self-regulation and attention are linked to better academic performances (Mrazek et al., 2013); and (3) Improve wellbeing and resilience: By promoting self-awareness and emotional regulation, mindfulness cultivates a calmer, more reflective approach to academic challenges (Shankland and Rosset, 2017).

Another domain is strengths-based learning, where students discover and cultivate their individual strengths, i.e., their creativity, leadership, or perseverance (Hodges and Clifton, 2004). Incorporating activities such as strengths inventories or reflective assignments connecting strengths to the course content can enhance motivation and engagement. Students that acknowledge and use their strengths during academic tasks often display higher intrinsic motivation (Ryan and Deci, 2020). It can also promote positive emotions and self-efficacy, that is, a strengths focus can change students’ mindsets from deficit-correction to growth and possibility (Proctor et al., 2011). In a similar vein, gratitude exercises (e.g., gratitude journaling or letter-writing) have been shown to be effective in increasing students’ subjective wellbeing and decreasing stress (Boehm et al., 2011). In the higher education context, short reflective activities or peer acknowledgment practices can promote a more supportive climate in the classrooms, which increases relatedness and student engagement.

### 4.2 Cultivating growth mindset for engagement and resilience

Previously described by Dweck (2006), the concept of growth mindset indicates that perceiving abilities to be changeable (rather than fixed) enables persistence, risk-taking, and resilience. Research shows that brief interventions designed to encourage a growth mindset—for example, writing materials that stress neuroplasticity or success stories showing the value of effort—can lead to substantial increases in academic performance (Yeager and Dweck, 2012). What higher education should look like as a whole is positive higher education, and growth-mindset practices can counter perfectionism and fear of failure, as well as help students begin to move toward adaptive coping strategies. When mistakes are normalized as learning opportunities, students might be more likely to ask for help and explore tough topics (Dweck, 2006). Growth mindset interventions can promote reappraisals of setbacks as information for improvement, not definitive judgments of one’s ability (Burnette et al., 2013).

Mindfulness interventions, strengths-based learning, gratitude exercises, and growth mindset programs are a few ways to achieve this through their alignment with SDT principles: they collectively support autonomy, competence and relatedness (Ryan, 2017). These interventions also assist students in developing internal resources (e.g., emotion-regulatory and self-reflective skills, optimistic thinking patterns) and external support structures (e.g., peer bonding, instructor guidance, inclusive class culture) that function to build resilience (Martin and Marsh, 2009). Students who perceive their learning context—their classroom, school, or workplace—as caring and inspiring are much more likely to remain focused, active, and motivated in their learning (Furlong et al., 2014).

Though there are proven benefits, implementing positive education strategies in higher education can be challenging because of logistical and cultural challenges (Oades et al., 2011). For example, while they may be well practiced at leading mindfulness or strengths-based activities, instructors may need professional development opportunities where they might feel more comfortable leading such activities in large lecture courses (Bamber and Kraenzle Schneider, 2016). In addition, Waters (2011) points out that the effectiveness of positive interventions is influenced by program duration and fidelity of implementation and integration into existing curriculum. Scattered brief exercises may lead to minimal effect, as opposed to systematic activity, potentially leading to a sedentary engagement, integrated within course content or co-curricular programs (Conley et al., 2016). Positive education approaches are built on the fundamental premise that resilience and engagement at the university level can be cultivated through mindfulness, strengths-based learning, gratitude and a growth mindset. If aligned with SDT (particularly the core tenets of autonomy, competence, and relatedness), these programs can contribute to a holistic educational experience centered around academic engagement, emotional and psychological health improvement.

### 4.3 Embedding wellbeing in institutional policies

Evidence suggests that higher education institutions need institutionalize wellbeing, as opposed to allocating wellbeing support to optional or peripheral programs (Oades et al., 2011). A genuinely wellbeing-centered pedagogy infuses campus culture, informing administrative policies, faculty development, and student support services (Kern et al., 2015). This change indicates a shift being prioritized by universities—not just student academic or career outcomes, but a holistic vision of student growth (Seligman, 2011). Institutions will then establish a systemic platform that sustains and magnifies positive education strategies over time by allowing wellbeing to integrate with mission statements, course objectives, professional training, and future needs. Features that capture department-level commitment to embedding wellbeing in institution-wide policies could include: (1) Curriculum Design: A requirement within departments that at least one module or assignment within core courses be related to wellbeing (Waters, 2011); (2) Assessment Measures: Standards beyond grades, whereby wellbeing measures [e.g., self-reported metrics of flourishing, belonging, and resilience can be included as indicators in program evaluations (Kern et al., 2015)]; and (3) Faculty Incentives: Encouraging and rewarding autonomy-supportive, strengths-based, and mindful teaching practices through tenure and promotion processes (Oades et al., 2011). By adopting the whole-university approach which includes supporting staff wellbeing as well as student wellbeing, such systemic changes, universities can create conditions in which positive education flourishes organically.

### 4.4 Leveraging technology for wellbeing

Preliminary evidence from systematic reviews indicates that mobile app-based psychological interventions can be feasible and yield positive outcomes for college students’ mental health (Oliveira et al., 2021), which suggests that technology can complement traditional counseling services, potentially alleviating some burden on overtaxed campus counseling centers. Another promising approach is to use digital platforms, possibly in conjunction with artificial intelligence (AI), to deliver or supplement positive interventions. Mindfulness exercises, gratitude prompts, or strengths-based reflections can be delivered at scale through online modules or smartphone app (Conley et al., 2016). These can be used in customizing learning experiences, enhancing the mental health in an educational environment or even to make academic administration more efficient. As an example, adaptive learning systems can customize educational resources based on the personal preferences and competence of the student, which promotes greater autonomy, motivation, and success in performance.

AI-based platforms can also support on-demand support and counseling through chatbots and virtual advisors, who can assist students with stress, anxiety and academic pressures (Nelekar et al., 2022). As a result, institutions can also use early intervention and risk identification methods that can allow them to intervene and address potential dropouts or mental health problems in time, through the analysis of data related to student performance and attitudes (Raja et al., 2024). AI can also help fortify mental resilience by identifying indications of digital burnout or enabling support exchanges in crisis periods (Prinsloo et al., 2024; Rezapour and Elmshaeuser, 2022).

Nonetheless, AI-enabled educational systems should be considered carefully when integrating into higher education systems by focusing on equity, privacy, and ethical use of student data (Hidayat and Kahar, 2024). Disparities by socioeconomic background, reliable internet access, and available digital devices can widen these educational gaps even more (Téllez et al., 2024). Hence, the developers and institutions need to reduce bias in AI algorithms, be transparent on how data is collected and used, and respect cultural context when providing mental health support. Institutions can create pathways that support academic integrity at its highest attainable level by embedding AI responsibility and collaborative policy creation into academic processes, ultimately delivering a transformative educational ecosystem.

### 4.5 Cross-cultural perspectives and faculty development

Wellbeing-centered pedagogies also need to be culturally responsive—responsive to how social norms, belief systems and family expectations shape students’ experiences of autonomy and resilience. With the growth in number of the international students’ enrolment, educational institutions must adapt efficaciousness positive educational interventions to different cultural backgrounds (Oades et al., 2011). For instance, mindfulness may need to be re-conceptualized for students unfamiliar with contemplative practices, while strengths-based initiatives may need to be “cultured” to be relevant and resonate with students (Waters, 2011). An inclusive approach means acknowledging potential socio-economic and linguistic barriers to program access and participation (Kahu and Nelson, 2018). Increasing the autonomy support and strengths orientation is a key step and requires investing in faculty development—providing educators with the knowledge and skills to implement autonomy-supportive and strengths-based pedagogies (Reeve, 2016). Professional development workshops on positive higher education principles, growth mindset interventions, and culturally responsive teaching can provide educators with the tools to embed wellbeing within the courses (Jang et al., 2010). Simultaneously, institutional leaders—including deans and department chairs—are critical to modeling supportive behaviors, fostering a “top-down” commitment to positive higher education (Oades et al., 2011).

Although pilot interventions and small-scale studies have shown promise for better engaging and supporting students, the challenge of embedding these practices across the institution remains (Kern et al., 2015). Sustainable programs need long-term funding, cooperation among multiple departments, ongoing evaluation of their impact, and adaptations to improve interventions. Furthermore, tracking student wellbeing metrics across semesters or even academic years can provide insight into the longitudinal effects of positive education initiatives (Furlong et al., 2014). An important step toward broader acceptance may be to embed wellbeing measures into existing accreditation or quality assurance frameworks, so that positive education is regarded not as an add-on, but as a fundamental indicator of educational quality. The futures of positive higher education rely on system-wide structural change—policies in positions of power that support wellbeing, the ethical and inclusion-focused use of technology and systems—and intercultural readiness. Such efforts need to be guided by trained, supportive faculty and committed leadership that values student flourishing as integral to academic excellence. With a system-wide perspective, universities can evolve pedagogies that impart knowledge and skills, as well as resilience, engagement and holistic wellbeing in diverse student bodies.

## 5 Conclusion and discussion

### 5.1 Summary

The increased attention to positive higher education reflects the central importance of wellbeing, engagement and resilience to students’ learning and development. These reviewed frameworks represent a holistic model of higher education that positions mental health and personal development as necessary companions to academic achievement (Seligman et al., 2009). Wellbeing-oriented pedagogies extend from lecture-based initiatives and interventions, to embedding wellbeing in institutional policies, curriculum design and campus culture (Oades et al., 2011). If strategic planning and resource allocation are focused on students’ psychosocial needs, universities can transform into an ecosystem within which positive practices are thriving in multiple layers (Kern et al., 2015). Higher education institutions can leverage AI technologies to personalize learning experiences for students, even offering targeted mental health support. Such integrated strategies could more effectively account for the intricacies of student learning and retention, as well as prepare them in the long run for the complexities of real-world professional and personal endeavors (Kahu and Nelson, 2018).

### 5.2 Challenges

As a narrative review, this study does not include systematic meta-analysis or statistical comparisons of intervention efficacy. The conclusions are based on qualitative synthesis rather than empirical data collection, which may affect generalizability. Furthermore, literature from various geographical and cultural contexts may have been underrepresented in this review due to the availability of literature.

Though positive higher education is promising, there still remain several challenges. Many successful programs identified in pilot community-based initiatives are small (Waters, 2011). To scale them up requires institutional buy-in, long-term funding, and careful evaluation of programs (Kern et al., 2015). Positive education strategies should be culturally responsive, given the different beliefs about autonomy, mental health or emotional expression held by diverse student populations. Professional development opportunities and administrative support are crucial (Oades et al., 2011). Although it may facilitate the use of more autonomy-supportive teaching or new wellbeing activities, it risks adding to faculty workload, especially in large classes or resource-deprived environments (Reeve and Jang, 2006). Digital and AI-driven tools can enhance personalized, individualized learning for students and offer tailored mental health services, but privacy, equitable access, and ethical use of student data are designated areas that must be carefully managed. These are complex issues that require the kind of systemic perspective that refrains from pit-falling innovation against respect for diverse student experience but also protection of academic rigor.

### 5.3 Recommendations

Universities should adopt the whole university approach and articulate wellbeing and student development as part of their core mission (Oades et al., 2011). By treating wellbeing as “everyone’s business,” institutions signal its importance and avoid siloed efforts. To promote a culture of wellbeing and engagement in higher education, institutions can adopt a coordinated set of practices across teaching, administration, and student services. Educators should embed small-scale positive interventions—such as mindfulness or gratitude exercises—into existing learning, while using autonomy-supportive methods and growth mindset principles to strengthen intrinsic motivation. Administrators can institutionalize wellbeing metrics within assessment systems, offer ongoing faculty development, and encourage inclusive approaches that meet diverse cultural and linguistic needs. At the same time, student support services should broaden mental health initiatives to account for well-being rather than reactiveness and build a sense of community and belonging through peer-mentoring or small group activities and periodically measure intervention effectiveness to develop better strategies. By emphasizing the importance of these efforts in combination, universities can foster a sustainable ecosystem that propels both academic and holistic wellbeing as interconnected objectives.

Future investigations should focus longitudinal and cross-cultural studies by employing the use of AI tools to assess long-term outcomes for faculty and staff, as well as the reciprocal benefits from these initiatives. By working together, educators, administrators, policymakers and researchers can create learning environments that enable students to succeed not just at university but in their careers and communities. Through a holistic perspective of success—where intellectual engagement, emotional stability, and societal connectedness mutually reinforce one another—positive higher education has the potential to change the narrative around what it means to thrive in academia. In doing so, higher education institutions have the potential to cultivate generations of graduates who are not only skilled and knowledgeable, but also resilient, empathetic, and fully equipped to contribute constructively in an ever-evolving, AI-driven society.

## References

[ref1] Aloia L. S. McTigue M. (2019). Buffering against sources of academic stress: the influence of supportive informational and emotional communication on psychological well-being. Commun. Res. Rep. 36, 126–135. doi: 10.1080/08824096.2019.1590191

[ref2] Bakker A. B. Costa P. L. (2014). Chronic job burnout and daily functioning: a theoretical analysis. Burn. Res. 1, 112–119. doi: 10.1016/j.burn.2014.04.003

[ref3] Bakker A. B. Demerouti E. Sanz-Vergel A. (2023). Job demands–resources theory: ten years later. Annu. Rev. Organ. Psych. Organ. Behav. 10, 25–53. doi: 10.1146/annurev-orgpsych-120920-053933

[ref4] Bakker A. B. Sanz Vergel A. I. Kuntze J. (2015). Student engagement and performance: a weekly diary study on the role of openness. Motiv. Emot. 39, 49–62. doi: 10.1007/s11031-014-9422-5

[ref5] Bamber M. D. Kraenzle Schneider J. (2016). Mindfulness-based meditation to decrease stress and anxiety in college students: a narrative synthesis of the research. Educ. Res. Rev. 18, 1–32. doi: 10.1016/j.edurev.2015.12.004

[ref6] Baumeister R. F. Leary M. R. (1995). The need to belong: desire for interpersonal attachments as a fundamental human motivation. Psychol. Bull. 117, 497–529. doi: 10.1037/0033-2909.117.3.497, PMID: 7777651

[ref7] Baumeister R. F. Leary M. R. (1997). Writing narrative literature reviews. Rev. Gen. Psychol. 1, 311–320. doi: 10.1037/1089-2680.1.3.311

[ref8] Bizumic B. Reynolds K. J. Turner J. C. Bromhead D. Subasic E. (2009). The role of the group in individual functioning: school identification and the psychological well-being of staff and students. Appl. Psychol. 58, 171–192. doi: 10.1111/j.1464-0597.2008.00387.x

[ref9] Boehm J. K. Lyubomirsky S. Sheldon K. M. (2011). A longitudinal experimental study comparing the effectiveness of happiness-enhancing strategies in Anglo Americans and Asian Americans. Cognit. Emot. 25, 1263–1272. doi: 10.1080/02699931.2010.541227, PMID: 21432648 PMC4394986

[ref10] Burnette J. L. O'Boyle E. H. VanEpps E. M. Pollack J. M. Finkel E. J. (2013). Mind-sets matter: a meta-analytic review of implicit theories and self-regulation. Psychol. Bull. 139, 655–701. doi: 10.1037/a0029531, PMID: 22866678

[ref11] Conley C. S. Durlak J. A. Shapiro J. B. Kirsch A. C. Zahniser E. (2016). A meta-analysis of the impact of universal and indicated preventive technology-delivered interventions for higher education students. Prev. Sci. 17, 659–678. doi: 10.1007/s11121-016-0662-3, PMID: 27225631

[ref12] Deci E. L. Ryan R. M. (2000). The "what" and "why" of goal pursuits: human needs and the self-determination of behavior. Psychol. Inq. 11, 227–268. doi: 10.1207/S15327965PLI1104_01

[ref13] Demerouti E. Bakker A. B. Nachreiner F. Schaufeli W. B. (2001). The job demands-resources model of burnout. J. Appl. Psychol. 86, 499–512. doi: 10.1037/0021-9010.86.3.499 11419809

[ref14] Dorri Sedeh S. Aghaei A. (2024). The effectiveness of PERMA model education on university students’ well-being. J. Educ. Health Promot. 13:338. doi: 10.4103/jehp.jehp_840_23, PMID: 39679036 PMC11639425

[ref15] Dweck C. S. (2006). Mindset: The new psychology of success. New York: Random House.

[ref16] Furlong M. J. Gilman R. Huebner E. S. (2014). Handbook of positive psychology in schools. New York, NY: Routledge.

[ref17] Govindji R. Linley P. A. (2007). Strengths use, self-concordance and well-being: implications for strengths coaching and coaching psychologists. Int. Coach. Psychol. Rev. 2, 143–153. doi: 10.53841/bpsicpr.2007.2.2.143

[ref18] Goyal M. Singh S. Sibinga E. M. Gould N. F. Rowland-Seymour A. Sharma R.. (2014). Meditation programs for psychological stress and well-being: a systematic review and meta-analysis. JAMA Intern. Med. 174, 357–368. doi: 10.1001/jamainternmed.2013.13018, PMID: 24395196 PMC4142584

[ref9001] Hendriks T. Schotanus-Dijkstra M. Hassankhan A. De Jong J. Bohlmeijer E. (2020). The efficacy of multi-component positive psychology interventions: A systematic review and meta-analysis of randomized controlled trials. J. Happiness Stud. 21, 357–390. doi: 10.1007/s10902-019-00082-1

[ref19] Hidayat A. Kahar M. R. (2024). Investigating the adoption of AI in higher education: a study of public universities in Indonesia. Cogent Educ. 11:2380175. doi: 10.1080/2331186X.2024.2380175

[ref20] Hodges T. D. Clifton D. O. (2004). Strengths-based development in practice. In Linley P. A. Joseph S. (Eds.), Positive psychology in practice (1, pp. 256–268). Wiley. Available online at: https://onlinelibrary.wiley.com/doi/10.1002/9780470939338.ch16

[ref21] Huppert F. A. So T. T. C. (2013). Flourishing across Europe: application of a new conceptual framework for defining well-being. Soc. Indic. Res. 110, 837–861. doi: 10.1007/s11205-011-9966-7, PMID: 23329863 PMC3545194

[ref22] Jang H. Reeve J. Deci E. L. (2010). Engaging students in learning activities: it is not autonomy support or structure but autonomy support and structure. J. Educ. Psychol. 102, 588–600. doi: 10.1037/a0019682

[ref23] Kabat-Zinn J. (2003). Mindfulness-based interventions in context: past, present, and future. Clin. Psychol. Sci. Pract. 10, 144–156. doi: 10.1093/clipsy.bpg016, PMID: 40927584

[ref24] Kahu E. R. Nelson K. (2018). Student engagement in the educational interface: understanding the mechanisms of student success. High. Educ. Res. Dev. 37, 58–71. doi: 10.1080/07294360.2017.1344197

[ref25] Kern M. L. Waters L. E. Adler A. White M. A. (2015). A multidimensional approach to measuring well-being in students: application of the PERMA framework. J. Posit. Psychol. 10, 262–271. doi: 10.1080/17439760.2014.936962, PMID: 25745508 PMC4337659

[ref26] Körner L. S. Mülder L. M. Bruno L. Janneck M. Dettmers J. Rigotti T. (2023). Fostering study crafting to increase engagement and reduce exhaustion among higher education students: a randomized controlled trial of the STUDYCoach online intervention. Appl. Psychol. Health Well Being 15, 776–802. doi: 10.1111/aphw.12410, PMID: 36261917

[ref27] Kovich M. K. Simpson V. L. Foli K. J. Hass Z. Phillips R. G. (2023). Application of the PERMA model of well-being in undergraduate students. Int. J. Community Well-Being 6, 1–20. doi: 10.1007/s42413-022-00184-4, PMID: 36320595 PMC9607835

[ref28] Lesener T. Pleiss L. S. Gusy B. Wolter C. (2020). The study demands-resources framework: an empirical introduction. Int. J. Environ. Res. Public Health 17:5183. doi: 10.3390/ijerph17145183, PMID: 32709128 PMC7400357

[ref29] Li H. (2025). Students’ wellbeing in positive higher education: conceptual frameworks and influencing factors. Front. Educ. 10:1607364. doi: 10.3389/feduc.2025.1607364

[ref30] Madigan D. J. Curran T. (2021). Does burnout affect academic achievement? A meta-analysis of over 100,000 students. Educ. Psychol. Rev. 33, 387–405. doi: 10.1007/s10648-020-09533-1

[ref31] Martin A. J. Ginns P. Collie R. J. (2023). University students in COVID-19 lockdown: the role of adaptability and fluid reasoning in supporting their academic motivation and engagement. Learn. Instr. 83:101712. doi: 10.1016/j.learninstruc.2022.101712, PMID: 36320939 PMC9613803

[ref32] Martin A. J. Marsh H. W. (2009). Academic resilience and academic buoyancy: multidimensional and hierarchical conceptual framing of causes, correlates and cognate constructs. Oxf. Rev. Educ. 35, 353–370. doi: 10.1080/03054980902934639

[ref33] McGrath H. Noble T. (2011). Bounce back!: a wellbeing and resilience program. Years 5-8. Melbourne: Deakin University.

[ref34] Miserandino M. (1996). Children who do well in school: individual differences in perceived competence and autonomy in above-average children. J. Educ. Psychol. 88, 203–214. doi: 10.1037/0022-0663.88.2.203

[ref35] Mokgele K. R. Rothmann S. (2014). A structural model of student well-being. S. Afr. J. Psychol. 44, 514–527. doi: 10.1177/0081246314541589

[ref36] Morgan B. Simmons L. (2021). A ‘PERMA’ response to the pandemic: an online positive education Programme to promote wellbeing in university students. Front. Educ. 6:642632. doi: 10.3389/feduc.2021.642632

[ref37] Mrazek M. D. Franklin M. S. Phillips D. T. Baird B. Schooler J. W. (2013). Mindfulness training improves working memory capacity and GRE performance while reducing mind wandering. Psychol. Sci. 24, 776–781. doi: 10.1177/0956797612459659, PMID: 23538911

[ref38] Nelekar S. Abdulrahman A. Gupta M. Richards D. (2022). Effectiveness of embodied conversational agents for managing academic stress at an Indian university (ARU) during COVID-19. Br. J. Educ. Technol. 53, 491–511. doi: 10.1111/bjet.13174

[ref39] Noble T. McGrath H. (2015). Prosper: a new framework for positive education. Psychol. Well-Being 5:2. doi: 10.1186/s13612-015-0030-2

[ref40] Oades L. G. Robinson P. Green S. Spence G. B. (2011). Towards a positive university. J. Posit. Psychol. 6, 432–439. doi: 10.1080/17439760.2011.634828

[ref41] Oliveira C. Pereira A. Vagos P. Nóbrega C. Gonçalves J. Afonso B. (2021). Effectiveness of Mobile app-based psychological interventions for college students: a systematic review of the literature. Front. Psychol. 12:647606. doi: 10.3389/fpsyg.2021.647606, PMID: 34045994 PMC8144454

[ref42] Osterman K. F. (2000). Students' need for belonging in the school community. Rev. Educ. Res. 70, 323–367. doi: 10.3102/00346543070003323

[ref43] Prinsloo P. Khalil M. Slade S. (2024). Vulnerable student digital well-being in AI-powered educational decision support systems (AI-EDSS) in higher education. Br. J. Educ. Technol. 55, 2075–2092. doi: 10.1111/bjet.13508

[ref44] Proctor C. Maltby J. Linley P. A. (2011). Strengths use as a predictor of well-being and health-related quality of life. J. Happiness Stud. 12, 153–169. doi: 10.1007/s10902-009-9181-2

[ref45] Raja S. Jebadurai D. J. Ivan L. Mykola R. V. Ruslan K. Nadiia P. R. (2024). “Impact of artificial intelligence in students’ learning life” in Studies in systems, decision and control, vol. 516. (Cham, Switzerland: Springer Science and Business Media Deutschland GmbH), 3–17.

[ref46] Reeve J. (2016). Autonomy-supportive teaching: what it is, how to do it. In Liu W. C. Wang J. C. K. Ryan R. M. (Eds.), Building autonomous learners (129–152). Springer Singapore. Available online at: https://link.springer.com/10.1007/978-981-287-630-0_7

[ref47] Reeve J. Jang H. (2006). What teachers say and do to support students' autonomy during a learning activity. J. Educ. Psychol. 98, 209–218. doi: 10.1037/0022-0663.98.1.209

[ref48] Reeve J. Tseng C.-M. (2011). Agency as a fourth aspect of students’ engagement during learning activities. Contemp. Educ. Psychol. 36, 257–267. doi: 10.1016/j.cedpsych.2011.05.002

[ref49] Reis H. T. Sheldon K. M. Gable S. L. Roscoe J. Ryan R. M. (2000). Daily well-being: the role of autonomy, competence, and relatedness. Personal. Soc. Psychol. Bull. 26, 419–435. doi: 10.1177/0146167200266002

[ref50] Rezapour M. Elmshaeuser S. K. (2022). Artificial intelligence-based analytics for impacts of COVID-19 and online learning on college students’ mental health. PLoS One 17:e0276767. doi: 10.1371/journal.pone.0276767, PMID: 36399458 PMC9674166

[ref51] Ryan R. M. (2017). Self-determination theory: Basic psychological needs in motivation, development, and wellness. New York: Guilford Press.

[ref52] Ryan R. M. Deci E. L. (2020). Intrinsic and extrinsic motivation from a self-determination theory perspective: definitions, theory, practices, and future directions. Contemp. Educ. Psychol. 61:101860. doi: 10.1016/j.cedpsych.2020.101860

[ref53] Salmela-Aro K. Upadyaya K. (2014). School burnout and engagement in the context of demands–resources model. Br. J. Educ. Psychol. 84, 137–151. doi: 10.1111/bjep.12018, PMID: 24547758

[ref54] Schaufeli W. B. Bakker A. B. (2004). Job demands, job resources, and their relationship with burnout and engagement: a multi-sample study. J. Organ. Behav. 25, 293–315. doi: 10.1002/job.248

[ref55] Seligman M. E. (2011). Flourish: A visionary new understanding of happiness and well-being. Sydney: Simon and Schuster.

[ref56] Seligman M. E. P. Ernst R. M. Gillham J. Reivich K. Linkins M. (2009). Positive education: positive psychology and classroom interventions. Oxf. Rev. Educ. 35, 293–311. doi: 10.1080/03054980902934563

[ref57] Shankland R. Rosset E. (2017). Review of brief school-based positive psychological interventions: a taster for teachers and educators. Educ. Psychol. Rev. 29, 363–392. doi: 10.1007/s10648-016-9357-3

[ref58] Téllez A. R. Ortiz L. M. F. Domínguez F. C. T. (2024). Artificial intelligence in university administration: an overview of its uses and applications. Rev. Interam. Bibl. 47:e353620. doi: 10.17533/udea.rib.v47n2e353620

[ref59] Vansteenkiste M. Simons J. Lens W. Sheldon K. M. Deci E. L. (2004). Motivating learning, performance, and persistence: the synergistic effects of intrinsic goal contents and autonomy-supportive contexts. J. Pers. Soc. Psychol. 87, 246–260. doi: 10.1037/0022-3514.87.2.246, PMID: 15301630

[ref60] Waters L. (2011). A review of school-based positive psychology interventions. Aust. Educ. Dev. Psychol. 28, 75–90. doi: 10.1375/aedp.28.2.75

[ref61] Wehmeyer M. L. Cheon S. H. Lee Y. Silver M. (2021). Self-determination in positive education. In Kern M. L. Wehmeyer M. L. (Eds.), The Palgrave handbook of positive education (225–249). Springer International Publishing. Available online at: https://link.springer.com/10.1007/978-3-030-64537-3_9

[ref62] Yeager D. S. Dweck C. S. (2012). Mindsets that promote resilience: when students believe that personal characteristics can be developed. Educ. Psychol. 47, 302–314. doi: 10.1080/00461520.2012.722805

